# Study of lipoprotein(a) and its impact on atherosclerotic cardiovascular disease: Design and rationale of the Mass General Brigham Lp(a) Registry

**DOI:** 10.1002/clc.23456

**Published:** 2020-09-06

**Authors:** Adam N. Berman, David W. Biery, Curtis Ginder, Olivia L. Hulme, Daniel Marcusa, Orly Leiva, Wanda Y. Wu, Avinainder Singh, Sanjay Divakaran, Jon Hainer, Alexander Turchin, James L. Januzzi, Pradeep Natarajan, Christopher P. Cannon, Marcelo F. Di Carli, Deepak L. Bhatt, Ron Blankstein

**Affiliations:** ^1^ Cardiovascular Division, Department of Medicine Brigham and Women's Hospital, Harvard Medical School Boston Massachusetts USA; ^2^ Department of Medicine Brigham and Women's Hospital, Harvard Medical School Boston Massachusetts USA; ^3^ Department of Medicine Yale University School of Medicine New Haven Connecticut USA; ^4^ Department of Radiology Brigham and Women's Hospital, Harvard Medical School Boston Massachusetts USA; ^5^ Division of Endocrinology, Department of Medicine Brigham and Women's Hospital, Harvard Medical School Boston Massachusetts USA; ^6^ Cardiology Division Massachusetts General Hospital, Harvard Medical School Boston Massachusetts USA

**Keywords:** ASCVD, lipoprotein(a), natural language processing, prevention

## Abstract

Lipoprotein(a) [Lp(a)] is independently associated with atherosclerotic cardiovascular disease and calcific aortic valve stenosis. Elevated Lp(a) affects approximately one in five individuals and meaningfully contributes to the residual cardiovascular risk in individuals with otherwise well‐controlled risk factors. With targeted therapies in the therapeutic pipeline, there is a need to further characterize the clinical phenotypes and outcomes of individuals with elevated levels of this unique biomarker. The Mass General Brigham Lp(a) Registry will be built from the longitudinal electronic health record of two large academic medical centers in Boston, Massachusetts, to develop a detailed cohort of patients who have had their Lp(a) measured. In combination with structured data sources, clinical documentation will be analyzed using natural language processing techniques to accurately characterize baseline characteristics. Important outcome measures including all‐cause mortality, cardiovascular mortality, and cardiovascular events will be available for analysis. Approximately 30 000 patients who have had their Lp(a) tested within the Mass General Brigham system from January 2000 to July 2019 will be included in the registry. This large Lp(a) cohort will provide meaningful observational data regarding the differential risk associated with Lp(a) values and cardiovascular disease. With a new frontier of targeted Lp(a) therapies on the horizon, the Mass General Brigham Lp(a) Registry will help provide a deeper understanding of Lp(a)'s role in long term cardiovascular outcomes.

## 1 INTRODUCTION

Over the last four decades, numerous studies have demonstrated that elevated lipoprotein(a) (Lp(a)) is an independent risk factor for coronary heart disease, ischemic stroke, and calcific aortic valve stenosis.^1^, ^2^, ^3^, ^4^, ^5^, ^6^, ^7^, ^8^, ^9^, ^10^, ^11^, ^12^ With a unique molecular structure hypothesized to be both pro‐atherogenic and pro‐thrombotic,^13^ Lp(a) is composed of a low‐density lipoprotein‐like moiety covalently bound to a genetically‐mediated apolipoprotein(a) molecule.^14^ Synthesized in the liver and mediated almost entirely by the *LPA* gene, elevated Lp(a) is the most common inherited dyslipidemia, affecting approximately one in five individuals.^15^ Lp(a) levels above 50 mg/dL (≈ 125 nmol/L) correspond to the 80th percentile in population‐based studies and is the threshold at which its impact on atherosclerotic cardiovascular disease (ASCVD) has been shown to become clinically meaningful.^10^, ^16^, ^17^ Even for individuals with otherwise well‐controlled risk factors, elevated levels of Lp(a) have a significant and independent effect on residual atherosclerotic cardiovascular risk.^18^

Despite the robust evidence linking elevated levels of Lp(a) to cardiovascular disease, there are currently no approved pharmacologic therapies directly targeting Lp(a). Presently, those with significantly elevated levels are counseled on overall risk reduction and—in certain scenarios—recommended to initiate statin therapy.^19^, ^20^, ^21^ While statin therapy has no direct effect on Lp(a) levels,^22^ its use in some individuals with elevated Lp(a) centers around overall ASCVD risk reduction. However, there are now a variety of promising therapeutics in development which may transform the management of this dyslipidemia and help mitigate this important cardiovascular risk factor.^12^, ^23^, ^24^, ^25^, ^26^

With the landscape of Lp(a) management potentially shifting in the coming decade, there is a need to develop a deeper understanding as to which subsets of patients may derive the most benefit from future targeted therapies. Accordingly, we set out to assess the demographics, clinical characteristics, and outcomes of a large cohort of patients (n ≈ 30 000) who have had their Lp(a) measured over the last 20 years to further understand the association between Lp(a) levels and cardiovascular disease.

## 2 STUDY OBJECTIVES

The objectives of this study are to: (a) describe the distribution of Lp(a) across a large cohort of patients with and without baseline cardiovascular disease stratified by sex and ethnic background; (b) determine the association between Lp(a) level and cause‐specific mortality; (c) determine the association between Lp(a) level and incidence of myocardial infarction and non‐hemorrhagic stroke; (d) determine the association of Lp(a) level and the age of ASCVD events; (e) determine the association between Lp(a) level and venous thromboembolism;^13^ (f) evaluate the association between elevated Lp(a) levels and imaging correlates for coronary artery disease; (g) evaluate the association between Lp(a) level and peripheral revascularization; (h) determine the association between Lp(a) level and progression of aortic stenosis and need for aortic valve replacement; (i) assess the impact of Lp(a) and recurrent ASCVD events; and (j) assess the interaction of sex and ethnic background on the above investigations.

## 3 METHODS

The Mass General Brigham Lp(a) Registry will be a retrospective cohort of approximately 30 000 patients who had their Lp(a) measured from 01 January 2000 to 07 January 2019 as part of routine clinical care. The Institutional Review Board at Mass General Brigham approved this study.

**FIGURE 1:**
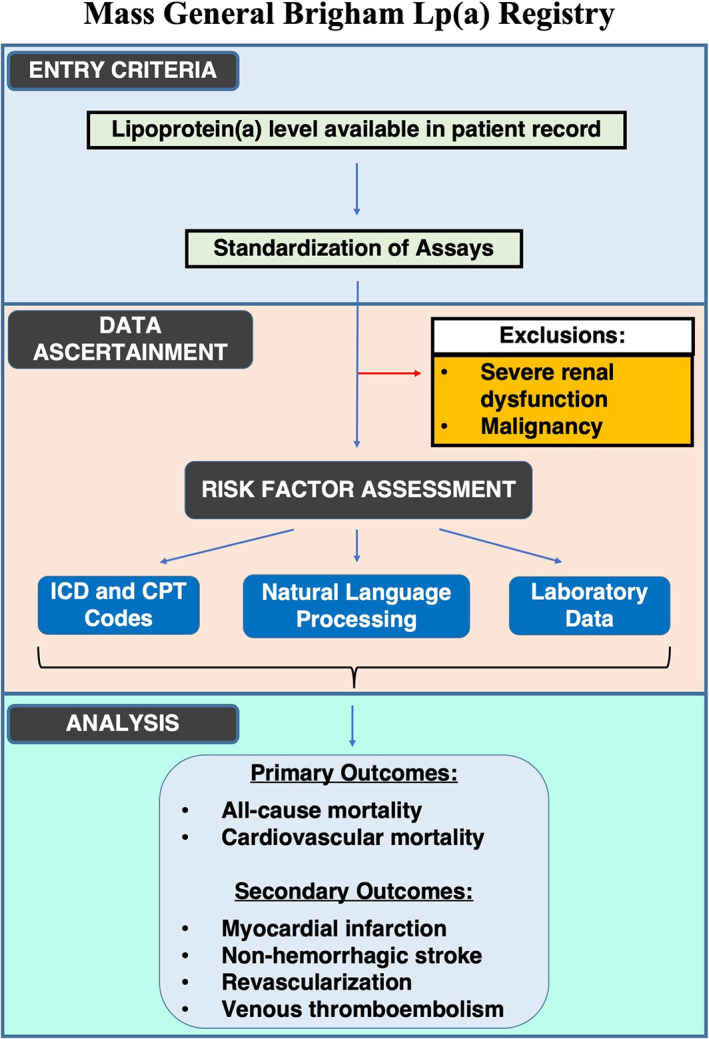
Design of the Mass General Brigham Lp(a) Registry. CPT, current procedural terminology; ICD, International Classification of Diseases; Lp(a), lipoprotein(a)

Figure 1 provides an outline of the study design.

### 3.1 Data sources

There are three primary data sources for our work:

1. The Research Patient Data Registry^27^ (RPDR) at Mass General Brigham will serve as the primary source of data for this registry. The RPDR is a centralized clinical data registry that consolidates clinical information from all hospitals within the Mass General Brigham network, including Brigham and Women's Hospital and Massachusetts General Hospital. This system provides demographic data, laboratory and imaging data, diagnostic and procedural codes, medication data, vital status, and clinical documentation for individuals meeting specified search criteria. RPDR obtains vital status from the Social Security Administration Death Master File.
2. Data from Medicare and Medicaid, when available, will be linked to individuals in our cohort to assess outcomes that may have occurred outside of the Mass General Brigham healthcare system.
3. International Classification of Diseases (ICD) coded death information from the National Death Index (NDI) or the Massachusetts Office of Vital Statistics will be used to determine the underlying and proximal causes of death for each patient who expired during the study period.

### 3.2 Entry criteria

All individuals ≥18 years of age with at least 1 Lp(a) result will be included in our cohort. There will be two exclusion criteria based on baseline covariates: (a) patients with a diagnosis of severe renal dysfunction defined as stage 5 chronic kidney disease (estimated glomerular filtration rate < 15 mL/min/m^2^), those who have had a renal transplant, or those on dialysis; and (b) individuals with a diagnostic ICD code for a malignant neoplasm, excluding nonmelanoma skin cancers.

### 3.3 Standardization of Lp(a) values

Over the study period, two distinct Lp(a) assay methodologies were used as part of routine medical care. The first methodology was the standard immunochemical‐based assay with reference ranges <30 mg/dL or < 75 nmol/L.^10^, ^28^, ^29^ The second assay type was a clinically validated electrophoretic assay measuring the cholesterol content of Lp(a) particles with a reference range < 3 mg/dL.^30^, ^31^ Prior analytic work has demonstrated that this electrophoretic cholesterol assay has a strong correlation with the standard immunochemical‐based assay, with correlation coefficients ranging from 0.89 to 0.96.^30^, ^31^

In order to avoid potential biases due to differences in testing modalities over the study period, percentile groups for each assay will be defined separately and then combined. This methodology has been performed previously in other large Lp(a) studies.^2^, ^5^, ^9^, ^32^ For selected analyses, if appropriate, we will maintain Lp(a) levels in their original form for use as continuous variables within individual assay types or testing methodologies.

### 3.4 Assessment of baseline covariates

Given the presence of more than 8 million clinical notes and tens of millions of structured data points (eg, laboratory values, ICD codes, and so on) relating to our study population, accurately characterizing all individuals in the cohort will be essential for analytic work.

#### 3.4.1 Methods for baseline covariate assessment

Baseline covariates will be determined through the following methods:

1. Demographic data, such as age, sex, and race, will be obtained from the Mass General Brigham RPDR system.
2. Natural language processing (NLP) algorithms using the open‐source Canary NLP platform will be created and applied to clinical documentation to determine the presence of a variety of key baseline risk factors.^33^, ^34^ Covariates assessed in this fashion will be the clinical diagnoses and documentation at any time of hypertension, dyslipidemia, diabetes, transient ischemic attack, nonhemorrhagic stroke, coronary artery disease, myocardial infarction, or coronary revascularization. Each of these algorithms will be individually validated for their accuracy prior to all analytic work.
3. Smoking status for each individual per calendar year in the cohort will be determined through the use of a previously validated NLP system.^35^ This NLP system has high accuracy and is able to assign an annual smoking status to each patient with a sensitivity of 92.4%, specificity of 86.2%, and AUC of 0.89. Because this system was developed using the Mass General Brigham hospital network RPDR database, it will be able to accurately assess for patient‐level smoking status within our registry.
4. Laboratory data will be used for further assessment of baseline covariates. Key laboratory data will include creatinine, glycated hemoglobin (HgbA1c), and the individual components of the complete cholesterol panel (eg, total cholesterol, triglycerides, high‐density lipoprotein cholesterol, and low‐density lipoprotein cholesterol).
  - Diabetes will be defined as a HgbA1c level ≥ 6.5%. For women under the age of 45, diabetes will be defined by the presence of two HgbA1c levels ≥6.5% separated by at least 9 months to avoid categorizing those with gestational diabetes as having a diagnosis of diabetes.
  - Dyslipidemia will be defined as total cholesterol ≥240 mg/dL, serum triglycerides ≥150 mg/dL, high‐density lipoprotein cholesterol <40 mg/dL in men or <50 mg/dL in women.
  - Chronic kidney disease will be defined as a median estimated glomerular filtration rate between 60 and 15 mL/min/m^2^.
5. Medication use, such as the use of cholesterol lowering therapies or the use of insulin therapy for those with diabetes, will be assessed using NLP as well as through analysis of electronic prescription data from the RPDR data repository.
6. Diagnostic and procedural ICD‐9, ICD‐10, and Current Procedural Terminology (CPT) codes will further augment baseline risk factor assessment. A full description of the covariates, which will be assessed using ICD and/or CPT codes is available in the Appendix S1.
7. Markers of socioeconomic status will be incorporated into our analyses using the Area Deprivation Index (ADI). The ADI is a validated marker of neighborhood level socioeconomic disadvantage, a surrogate for individual socioeconomic status. The ADI combines 17 measures of employment, income, housing, and education from the American Community Survey to create a score for each geographic unit in the United States.^36^, ^37^, ^38^, ^39^, ^40^, ^41^, ^42^ Each patient's home address will be indexed to its associated neighborhood level socioeconomic disadvantage ADI score and incorporated into multivariate analyses.

#### 3.4.2 Baseline covariate assessment period

The baseline covariate assessment period will be determined in one of two ways depending on the analysis:

1. Based on Lp(a) testing date: Baseline covariates will be assessed up to 12 months prior to and 12 months after the date of Lp(a) laboratory testing. For individuals with multiple Lp(a) tests over time, the baseline covariate assessment period will relate to the first chronologic Lp(a) measurement.
2. Based on first encounter: Baseline covariates will be assessed from the date of the first clinical encounter in the RPDR system and up to 24 months thereafter.

Additionally, the use of specific medications will be determined based on data 12 months prior to and up to 6 months after the date of Lp(a) laboratory testing or up to 12 months after the first encounter in the RPDR system. From information available during the covariate assessment period, we will calculate baseline 10‐year ASCVD risk for individuals without a history of myocardial infarction or stroke.^20^, ^21^, ^43^

### 3.5 Outcomes and index date for follow‐up

The key outcome measures in our study will be all‐cause mortality, cardiovascular mortality, and the occurrence of cardiovascular events. Follow‐up for the study outcomes will commence after the baseline covariate assessment period.

#### 3.5.1 All‐cause mortality

Vital status will be determined from the NDI and the Massachusetts Office of Vital Statistics in conjunction with data from the Social Security Administration Death Master File.

#### 3.5.2 Cardiovascular mortality

Cardiovascular mortality will be determined based on the ICD‐coded underlying cause of death^44^, ^45^, ^46^, ^47^ as determined by the NDI or the Massachusetts Office of Vital Statistics. When applicable, cardiovascular disease codes listed as a contributing factor in noncardiovascular deaths will be used in sensitivity analyses to estimate the potential additional effect that overall cardiovascular disease has on all‐cause mortality.

#### 3.5.3 Cardiovascular events

For cardiovascular events requiring hospitalization, such as acute myocardial infarction, unstable angina, venous thromboembolism, nonhemorrhagic stroke, or transient ischemic attack, the presence of a diagnostic ICD code in the primary hospital discharge position will be considered diagnostic. This methodology has been extensively validated with high specificity, high positive predictive value, and reasonable sensitivity.^48^, ^49^, ^50^, ^51^, ^52^, ^53^, ^54^, ^55^, ^56^, ^57^ When applicable, we will perform sensitivity analyses incorporating the same diagnostic codes as indicating the presence of a given outcome when they appear in nonprimary positions in hospital discharge claims data.^52^, ^56^

For outcomes, such as coronary revascularization, peripheral revascularization, transcatheter or surgical aortic valve replacement, and other relevant cardiovascular procedures, a single procedural code in the patient record (either ICD or CPT) will be considered diagnostic.

Linked Medicare and Medicaid claims data (where available) will be used to supplement outcomes data for individuals who received medical care for acute events and procedures outside of our hospital network. Sensitivity analyses will be performed to compare the incidence of cardiovascular events in our hospital system vs those that occurred in other systems.

### 3.6 Data management

Study‐related data for all patients who meet inclusion criteria will be stored on secure servers hosted by Mass General Brigham. Project managers (the primary investigator, analytic team, and data steward) will determine which members of the research team are authorized to access file system data. As part of research orientation at Mass General Brigham, all users are required to complete privacy, best practice, and ethics training provided by the Collaborative Institutional Training Initiative (www.citiprogram.org). Where appropriate, institutional data use agreements will be orchestrated to facilitate the research enterprise.

#### 3.6.1 Statistical analysis

Clinical characteristics will be compared across different subgroups, including sex and ethnic background. Continuous variables will be reported as means or medians and compared with *t* tests, rank‐sum tests, or analysis of variance, as appropriate. Categorical variables will be reported as frequencies and proportions and will be compared with *χ* ^2^ or Fisher exact tests. Ordinal variables will be compared using an appropriate trend test. Analyses will be adjusted using regression techniques for available baseline covariates to adjust for confounding and to examine associations between Lp(a) level and the outcomes of interest. Cox proportional hazards modeling will be performed for time‐to‐event analyses. Incidence rate ratios will be calculated when comparing the rates of cardiovascular events between different levels of Lp(a). All analyses will be performed on deidentified data.

## 4 LIMITATIONS

As a retrospective cohort study spanning a 20‐year period, we are limited by differences in the techniques used for Lp(a) measurement. However, to address this, we will standardize each assay based on percentiles and subsequently perform analyses across predefined percentile groups.^2^, ^5^, ^9^, ^32^ Additionally, our cohort consists of patients who were tested as part of routine clinical care, which may introduce selection bias. However, at present, Lp(a) testing is typically performed on selected patients with cardiovascular disease. As such, our findings will be applicable to patients who are tested in the current era where Lp(a) screening is typically performed in those at elevated cardiovascular risk while also providing meaningful information for the vast majority of patients with elevated levels.

Given the retrospective nature of this work, unmeasured and residual confounding may still exist even after adjusting for known confounders. Because this study is limited to two large academic medical centers in one geographic region of the United States, the results may not be fully generalizable to other regions or practice environments. Finally, some nonfatal endpoints (eg, myocardial infarction, stroke, and so on) that occurred outside of our healthcare system or were not captured by Medicare and Medicaid claims data may be missed. However, we do not expect that these missed events will significantly bias our results as an individual's Lp(a) level should not impact whether they seek care for an acute event within a given medical system (eg, nondifferential data loss). Additionally, analyses based on all‐cause and cardiovascular mortality are expected to have complete information through national vital status and cause of death data repositories, thereby limiting the possibility of data loss affecting our findings.

Nevertheless, there are multiple important strengths to our study. Our registry will include a large number of patients, making it one of the largest Lp(a) registries with one of the longest follow‐up periods in the United States. Via linkage with Medicare and Medicaid claims data, we will be able to assess for events that occurred outside of our hospital network, thus leveraging the advantages of our detailed patient level information with the power of national claims data. Finally, our study data will be based on a combination of detailed clinical notes, laboratory values, and billing records, which will provide a high degree of accuracy and detail that would not be possible when relying on administrative data alone. Moreover, we will use innovative natural language processing algorithms to adjudicate baseline variables from clinical documentation, a technique which provides highly accurate patient‐level data.^33^, ^58^, ^59^, ^60^

## 5 DISCUSSION

Important work linking Lp(a) with cardiovascular disease combined with the ongoing development of targeted therapies have created a resurgence of interest in this unique lipoprotein. Current ongoing trials^23^, ^24^ of antisense oligonucleotides and the development of small interfering RNAs (siRNAs)^61^ will elucidate the effect of such therapies and their impact on incident ASCVD.

Given the heterogenous level of risk associated with elevated levels of Lp(a), large observational studies are important for further informing the differential risk associated with Lp(a) values and cardiovascular disease. Additionally, developing a deeper understanding of how Lp(a) interacts with other established risk factors will be valuable in refining cardiovascular risk prediction. The Mass General Brigham Lp(a) Registry will provide real‐world data to help inform these important questions. Additionally, this registry may help identify subgroups of patients who may benefit the most from pharmacologic intervention as therapies emerge. Finally, given the large size of our cohort as well as the use of robust methods to determine baseline characteristics and outcomes, our study will provide patient level data that can be used to inform future clinical trials.

## CONFLICT OF INTEREST

Dr D. L. B. discloses the following relationships‐Advisory Board: Cardax, Cereno Scientific, Elsevier Practice Update Cardiology, Medscape Cardiology, PhaseBio, PLx Pharma, Regado Biosciences; Board of Directors: Boston VA Research Institute, Society of Cardiovascular Patient Care, TobeSoft; Chair: American Heart Association Quality Oversight Committee; Data Monitoring Committees: Baim Institute for Clinical Research (formerly Harvard Clinical Research Institute, for the PORTICO trial, funded by St. Jude Medical, now Abbott), Cleveland Clinic (including for the ExCEED trial, funded by Edwards), Duke Clinical Research Institute, Mayo Clinic, Mount Sinai School of Medicine (for the ENVISAGE trial, funded by Daiichi Sankyo), Population Health Research Institute; Honoraria: American College of Cardiology (Senior Associate Editor, Clinical Trials and News, ACC.org; Vice‐Chair, ACC Accreditation Committee), Baim Institute for Clinical Research (formerly Harvard Clinical Research Institute; RE‐DUAL PCI clinical trial steering committee funded by Boehringer Ingelheim; AEGIS‐II executive committee funded by CSL Behring), Belvoir Publications (Editor in Chief, Harvard Heart Letter), Duke Clinical Research Institute (clinical trial steering committees, including for the PRONOUNCE trial, funded by Ferring Pharmaceuticals), HMP Global (Editor in Chief, Journal of Invasive Cardiology), Journal of the American College of Cardiology (Guest Editor; Associate Editor), Medtelligence/ReachMD (CME steering committees), Population Health Research Institute (for the COMPASS operations committee, publications committee, steering committee, and USA national co‐leader, funded by Bayer), Slack Publications (Chief Medical Editor, Cardiology Today's Intervention), Society of Cardiovascular Patient Care (Secretary/Treasurer), WebMD (CME steering committees); Other: Clinical Cardiology (Deputy Editor), NCDR‐ACTION Registry Steering Committee (Chair), VA CART Research and Publications Committee (Chair); Research Funding: Abbott, Afimmune, Amarin, Amgen, AstraZeneca, Bayer, Boehringer Ingelheim, Bristol‐Myers Squibb, Cardax, Chiesi, CSL Behring, Eisai, Ethicon, Ferring Pharmaceuticals, Forest Laboratories, Fractyl, Idorsia, Ironwood, Ischemix, Lexicon, Lilly, Medtronic, Pfizer, PhaseBio, PLx Pharma, Regeneron, Roche, Sanofi Aventis, Synaptic, The Medicines Company; Royalties: Elsevier (Editor, Cardiovascular Intervention: A Companion to Braunwald's Heart Disease); Site Co‐Investigator: Biotronik, Boston Scientific, CSI, St. Jude Medical (now Abbott), Svelte; Trustee: American College of Cardiology; Unfunded Research: FlowCo, Merck, Novo Nordisk, Takeda. Dr R. B. received research support from Amgen Inc and Astellas Inc. Dr C. P. C. repots research grants from Amgen, Boehringer‐Ingelheim (BI), Bristol‐Myers Squibb (BMS), Daiichi Sankyo, Janssen, Merck, Pfizer. Additionally, he reports consulting fees from Aegerion, Alnylam, Amarin, Amgen, Applied Therapeutics, Ascendia, BI, BMS, Corvidia, HLS Therapeutics, Innovent, Janssen, Kowa, Merck, Pfizer, Sanofi, Rhoshan. Dr P. N. reports grant support from Amgen, Apple, and Boston Scientific, and is a scientific advisor to Apple and Blackstone Life Sciences. Dr A. T. reports having equity in Brio Systems and research funding from Astra Zeneca, Eli Lilly, Edwards, Novo Nordisk and Sanofi.

Sources of Funding: Dr. Berman is supported by a T32 postdoctoral training grant from the National Heart, Lung, and Blood Institute (T32 HL094301). Dr. Divakaran is supported by a T32 postdoctoral training grant from the National Heart, Lung, and Blood Institute (T32 HL094301).

## Supporting information


**Appendix S1.** Supporting Information.

## Data Availability

Data sharing not applicable to this article as no datasets were generated or analysed during the current study.
