# Supplementary material for: Study of lipoprotein(a) and its impact on atherosclerotic cardiovascular disease: Design and rationale of the Mass General Brigham Lp(a) Registry
Source: Clin Cardiol. 2020 Sep 6;43(11):1209–15. doi: 10.1002/clc.23456 (PMC7661644; doi:10.1002/clc.23456)
Supplement: Supplementary file 1 — Appendix S1. Supporting Information. [file CLC-43-1209-s001.docx]

| **Outcome Measure:** | **Definition:** | **Sources:** |
| --- | --- | --- |
| Acute Myocardial Infarction | A single ICD-9 or ICD-10 diagnosis code in the **primary** discharge position ^1-3^ | ICD-9 codes adapted from: ^1,3-6^  ICD-10 codes adapted from: ^4,7^ |
| Unstable Angina | A single ICD-9 or ICD-10 diagnosis code in the **primary** discharge position | ICD-9 codes adapted from: ^5,6^  ICD-10 codes adapted from: ^7^ |
| Ischemic Stroke | A single ICD-9 or ICD-10 diagnosis code in the **primary** discharge position ^6,8-10^ | ICD-9 codes adapted from: ^6,9,10^  ICD-10 codes adapted from: ^7,11^ |
| Transient Ischemic Attack | A single ICD-9 or ICD-10 diagnosis code in the **primary** discharge position ^9^ | ICD-9 codes adapted from: ^5,9^  ICD-10 codes adapted from: ^7^ |
| Percutaneous coronary intervention/ Angioplasty | A single ICD-9, ICD-10, CPT, or HCPCS procedure code | ICD-9 codes adapted from: ^6,12,13^  ICD-10 codes adapted from: ^14^  CPT Procedure codes from: ^6,15^ |
| Coronary Artery Bypass Grafting (CABG) | A single ICD-9, ICD-10, or CPT procedure code | ICD-9 codes adapted from: ^6,12,13^  ICD-10 codes adapted from: ^14^  CPT Procedure codes from: ^6,13,15^ |
| Acute Deep Venous Thrombosis | A single ICD-9 or ICD-10 diagnosis code in the **primary** hospital discharge position ^9,16^ | ICD-9 codes adapted from: ^9,10,16-18^  ICD-10 codes adapted from: ^7,17^ |
| Acute Pulmonary Embolism | A single ICD-9 or ICD-10 diagnosis code in the **primary** hospital discharge position ^9,16^ | ICD-9 codes adapted from: ^5,18^  ICD-10 codes adapted from: ^7^ |
| Aortic valve replacement (surgical or percutaneous) | A single ICD-9, ICD-10, or CPT procedure code | ICD-9 codes adapted from: ^12,19^  ICD-10 codes adapted from: ^14,19^ |
| Vascular dementia | A single ICD-9 or ICD-10 code, in any position, inpatient or outpatient | ICD-9 codes adapted from: ^5^  ICD-10 codes adapted from: ^7^ |
| Carotid endarterectomy or stenting | A single ICD-9, ICD-10, or CPT procedure code | ICD-9 codes adapted from: ^5,12,20,21^  ICD-10 codes adapted from: ^14^  CPT Procedure codes from: ^20^ |
| Peripheral revascularization or related procedure (e.g., amputation, etc.) | A single ICD-9, ICD-10, or CPT procedure code | ICD-9 codes adapted from: ^6,12,22,23^  ICD-10 codes adapted from: ^14^  CPT Procedure codes from: ^6,15,22,23^ |

| **Risk factor:** | **Definition:** | **Source:** |
| --- | --- | --- |
| Hypertension | At least one ICD-9 or ICD-10 codes, in any position, inpatient or outpatient | ICD-9 codes adapted from: ^10,24^  ICD-10 codes adapted from: ^7,24^ |
| Chronic kidney disease | At least one ICD-9, ICD-10, or CPT codes in any position, inpatient or outpatient | ICD-9 codes adapted from: ^5,10,25-27^  ICD-10 codes adapted from: ^7,25^ |
| Heart failure | A single ICD-9 or ICD-10 diagnosis code in the **primary** discharge position^28^ | ICD-9 codes adapted from: ^5,6,10,28^  ICD-10 codes adapted from: ^7,28^ |
| Atrial fibrillation / atrial flutter | At least one ICD-9 or ICD-10 code, in any position, inpatient or outpatient^10^ | ICD-9 codes adapted from: ^5^  ICD-10 codes adapted from: ^7^ |
| Type I diabetes | At least one ICD-9 or ICD-10 codes, in any position, inpatient or outpatient | ICD-9 codes adapted from: ^5^  ICD-10 codes adapted from: ^7^ |
| Type II diabetes | At least one ICD-9 or ICD-10 codes, in any position, inpatient or outpatient | ICD-9 codes adapted from: ^5^  ICD-10 codes adapted from: ^7^ |
| Smoking (cigarette) status | At least one ICD-9, ICD-10, or CPT code, in any position, inpatient or outpatient | ICD-9 codes adapted from: ^5^  ICD-10 codes adapted from: ^7^ |
| Cocaine use | At least one ICD-9 or ICD-10 code, in any position, inpatient or outpatient | ICD-9 codes adapted from: ^5^  ICD-10 codes adapted from: ^7^ |
| History of CAD | Composite measure, adapted from outcome section above |  |
| History of peripheral vascular disease | Composite measure, adapted from outcome section above |  |
| Cancer *  ** excluding non-melanoma skin cancer* | A single ICD-9 or ICD-10 codes, in any position, inpatient or outpatient | ICD-9 codes adapted from: ^29-31^  ICD-10 codes adapted from: ^7,29^ |
| End stage renal disease | At least one ICD-9, ICD-10, or CPT code, in any position, inpatient or outpatient | ICD-9 codes adapted from: ^2,32,33^  ICD-10 codes adapted from: ^32^  CPT codes adapted from: ^32,33^ |

**References:**

1. Kiyota Y, Schneeweiss S, Glynn RJ, Cannuscio CC, Avorn J, Solomon DH. Accuracy of Medicare claims-based diagnosis of acute myocardial infarction: estimating positive predictive value on the basis of review of hospital records. *Am Heart J.* 2004;148(1):99-104.

2. Initiative S. Mini-Sentinel Medical Product Assessment: a protocol for active surveillance of acute myocardial infarction in association with use of anti-diabetic agents. Anti-Diabetes Drugs and Acute Myocardial Infarction, Hospitalized Heart Failure Web site. <https://www.sentinelinitiative.org/sites/default/files/Drugs/Assessments/Mini-Sentinel_AMI-and-Anti-Diabetic-Agents_Protocol_0.pdf>. Published 2016. Updated 01/27/2016. Accessed 10/16/2019.

3. Yeh RW, Sidney S, Chandra M, Sorel M, Selby JV, Go AS. Population trends in the incidence and outcomes of acute myocardial infarction. *N Engl J Med.* 2010;362(23):2155-2165.

4. Initiative S. Coding Trend Analysis: Acute Myocardial Infarction. <https://www.sentinelinitiative.org/sites/default/files/surveillance-tools/validations-literature/AMI_Sentinel_Final_Report.pdf>. Published 2018. Accessed 10/16/2019.

5. Agency for Healthcare Research and Quality R, MD. HCUP CCS. Healthcare Cost and Utilization Project (HCUP). <www.hcup-us.ahrq.gov/toolssoftware/ccs/ccs.jsp>. Published 2017. Updated 03/2017. Accessed 10/03/2019.

6. Patorno E, Goldfine AB, Schneeweiss S, et al. Cardiovascular outcomes associated with canagliflozin versus other non-gliflozin antidiabetic drugs: population based cohort study. *BMJ.* 2018;360:k119.

7. Agency for Healthcare Research and Quality R, MD. Clinical Classifications Software Refined (CCSR) for ICD-10-CM Diagnoses. Healthcare Cost and Utilization Project (HCUP). <www.hcup-us.ahrq.gov/toolssoftware/ccsr/ccs_refined.js>. Published 2019. Updated 09/2019. Accessed 10/03/2019.

8. Andrade SE, Harrold LR, Tjia J, et al. A systematic review of validated methods for identifying cerebrovascular accident or transient ischemic attack using administrative data. *Pharmacoepidemiol Drug Saf.* 2012;21 Suppl 1:100-128.

9. Birman-Deych E, Waterman AD, Yan Y, Nilasena DS, Radford MJ, Gage BF. Accuracy of ICD-9-CM codes for identifying cardiovascular and stroke risk factors. *Med Care.* 2005;43(5):480-485.

10. Wang SV, Franklin JM, Glynn RJ, Schneeweiss S, Eddings W, Gagne JJ. Prediction of rates of thromboembolic and major bleeding outcomes with dabigatran or warfarin among patients with atrial fibrillation: new initiator cohort study. *BMJ.* 2016;353:i2607.

11. Initiative S. Ischemic Stroke Algorithm Defined in "Stroke, Gastrointestinal Bleeding, and Intracranial Hemorrhage following Apixaban or Warfarin Use in Patients with Non-Valvular Atrial Fibrillation: A Propensity Score Matched Analysis". <https://www.sentinelinitiative.org/sites/default/files/surveillance-tools/validations-literature/Ischemic_Stroke_apixaban_final_codelist.pdf>. Published 2019. Accessed 10/16/2019.

12. Agency for Healthcare Research and Quality R, MD. Procedure Classes for ICD-9-CM. <https://www.hcup-us.ahrq.gov/toolssoftware/procedure/procedure.jsp>. Accessed 05/20/2020.

13. Chang TI, Leong TK, Boothroyd DB, Hlatky MA, Go AS. Acute kidney injury after CABG versus PCI: an observational study using 2 cohorts. *J Am Coll Cardiol.* 2014;64(10):985-994.

14. Agency for Healthcare Research and Quality R, MD. Clinical Classifications Software (CCS) for ICD-10-PCS (beta version). <https://www.hcup-us.ahrq.gov/toolssoftware/ccs10/ccs10.jsp#download>. Published 2020. Accessed 05/21/2020.

15. CPT (Current Procedural Terminology) 4: Use the Current Procedural Terminology (CPT®) code set to bill outpatient & office procedures. <https://www.ama-assn.org/amaone/cpt-current-procedural-terminology>. Accessed 05/22/2020.

16. Tamariz L, Harkins T, Nair V. A systematic review of validated methods for identifying venous thromboembolism using administrative and claims data. *Pharmacoepidemiol Drug Saf.* 2012;21 Suppl 1:154-162.

17. Initiative S. Coding Trend Analysis: Deep Vein Thrombosis. <https://www.sentinelinitiative.org/sites/default/files/surveillance-tools/validations-literature/Deep_Vein_Thrombosis_Trend_Report.pdf>. Published 2019. Updated 08/21/2019. Accessed 10/17/2019.

18. White RH, Garcia M, Sadeghi B, et al. Evaluation of the predictive value of ICD-9-CM coded administrative data for venous thromboembolism in the United States. *Thrombosis Research.* 2010;126(1):61-67.

19. Hirji S, McGurk S, Kiehm S, et al. Utility of 90-Day Mortality vs 30-Day Mortality as a Quality Metric for Transcatheter and Surgical Aortic Valve Replacement Outcomes. *JAMA Cardiol.* 2019.

20. Kumamaru H, Jalbert JJ, Nguyen LL, et al. Surgeon case volume and 30-day mortality after carotid endarterectomy among contemporary medicare beneficiaries: before and after national coverage determination for carotid artery stenting. *Stroke.* 2015;46(5):1288-1294.

21. Jalbert JJ, Nguyen LL, Gerhard-Herman MD, et al. Comparative Effectiveness of Carotid Artery Stenting Versus Carotid Endarterectomy Among Medicare Beneficiaries. *Circ Cardiovasc Qual Outcomes.* 2016;9(3):275-285.

22. Nehler MR, Duval S, Diao L, et al. Epidemiology of peripheral arterial disease and critical limb ischemia in an insured national population. *J Vasc Surg.* 2014;60(3):686-695 e682.

23. Jaff MR, Cahill KE, Yu AP, Birnbaum HG, Engelhart LM. Clinical outcomes and medical care costs among medicare beneficiaries receiving therapy for peripheral arterial disease. *Ann Vasc Surg.* 2010;24(5):577-587.

24. Quan H, Khan N, Hemmelgarn BR, et al. Validation of a case definition to define hypertension using administrative data. *Hypertension.* 2009;54(6):1423-1428.

25. Initiative S. Coding Trend Analyses: Kidney Disease. <https://www.sentinelinitiative.org/sentinel/surveillance-tools/validations-lit-review/coding-trend-analyses-kidney-disease>. Published 2019. Updated 09/27/2019. Accessed 10/03/2019.

26. Wang SV, Huybrechts KF, Fischer MA, et al. Generalized boosted modeling to identify subgroups where effect of dabigatran versus warfarin may differ: An observational cohort study of patients with atrial fibrillation. *Pharmacoepidemiol Drug Saf.* 2018;27(4):383-390.

27. Winkelmayer WC, Schneeweiss S, Mogun H, Patrick AR, Avorn J, Solomon DH. Identification of individuals with CKD from Medicare claims data: a validation study. *Am J Kidney Dis.* 2005;46(2):225-232.

28. Saczynski JS, Andrade SE, Harrold LR, et al. A systematic review of validated methods for identifying heart failure using administrative data. *Pharmacoepidemiol Drug Saf.* 2012;21 Suppl 1:129-140.

29. Gagne JJ, Glynn RJ, Avorn J, Levin R, Schneeweiss S. A combined comorbidity score predicted mortality in elderly patients better than existing scores. *J Clin Epidemiol.* 2011;64(7):749-759.

30. Elixhauser A, Steiner C, Harris DR, Coffey RM. Comorbidity Measures for Use with Administrative Data. *Medical Care.* 1998;36(1):8-27.

31. Deyo R. Adapting a clinical comorbidity index for use with ICD-9-CM administrative databases. *Journal of Clinical Epidemiology.* 1992;45(6):613-619.

32. Initiative S. Coding Trend Analysis: Dialysis. <https://www.sentinelinitiative.org/sentinel/surveillance-tools/validations-lit-review/coding-trend-analyses-dialysis>. Published 2019. Accessed 11/04/2019.

33. Dave CV, Schneeweiss S, Kim D, Fralick M, Tong A, Patorno E. Sodium-Glucose Cotransporter-2 Inhibitors and the Risk for Severe Urinary Tract Infections: A Population-Based Cohort Study. *Ann Intern Med.* 2019;171(4):248-256.
